# Whip Use by Jockeys in a Sample of Australian Thoroughbred Races—An Observational Study

**DOI:** 10.1371/journal.pone.0033398

**Published:** 2012-03-19

**Authors:** Paul D. McGreevy, Robert A. Corken, Hannah Salvin, Celeste M. Black

**Affiliations:** 1 Faculty of Veterinary Science, University of Sydney, Sydney, New South Wales, Australia; 2 Faculty of Law, University of Sydney, Sydney, New South Wales, Australia; Indiana University, United States of America

## Abstract

The use of whips by jockeys is an issue. The current study viewed opportunistic high-speed footage of 15 race finishes frame-by-frame to examine the outcomes of arm and wrist actions (n = 350) on 40 horses viewed from the left of the field. Any actions fully or partially obscured by infrastructure or other horses were removed from the database, leaving a total of 104 non-contact sweeps and 134 strikes. For all instances of arm actions that resulted in fully visible whip strikes behind the saddle (n = 109), the outcomes noted were area struck, percentage of unpadded section making contact, whether the seam made contact and whether a visible indentation was evident on impact. We also recorded use of clockwise or counter-clockwise arm action from each jockey's whip, whether the whip was held like a tennis racquet or a ski pole, whether the hind leg on the side of the impact was in stance or swing phase and whether the jockey's arm was seen traveling above shoulder height. The goal of the study was to characterize the area struck and the visual impact of whip use at the level of the horse. We measured the ways in which both padded and unpadded sections of the whip made impact. There was evidence of at least 28 examples, in 9 horses, of breaches of the whip rules (one seam contact, 13 contacts with the head, and 14 arm actions that rose above the height of the shoulder). The whip caused a visible indentation on 83% of impacts. The unpadded section of the whip made contact on 64% of impacts. The results call into question the ability of Stewards to effectively police the rules concerning whip use and, more importantly, challenge the notion that padding the distal section of whips completely safeguards horses from any possible whip-related pain.

## Introduction

Whipping tired horses in the name of sport is becoming increasingly difficult to justify [1]. This view is supported by recent evidence showing that, in races of 1200 m and 1250 m, whip use was most frequent in the final two 200 m sections when horses were fatigued [2]. Further analysis of the same dataset revealed that horses were more likely to be whipped in the penultimate 200 m section of races if ridden by apprentice rather than non-apprentice jockeys and if drawn closer to the rail [3]. Given that horses further from the rail are known to have slower race times [4], one would expect them to need more whipping (if, indeed, whipping helps) to keep horses closer to the inside of the bend [3]. In a similar vein, the effectiveness of the whip in steering racehorses has been brought into doubt, in NSW at least, by data showing that handedness of riders, rather than direction of racing, seems to be the primary driver as to which hand jockeys use to carry the whip [5].

In Australia, the Australian Racing Board (ARB), representing the Thoroughbred racing industry, has developed the Australian Rules of Racing, and these rules are then adopted by state racing authorities, such as Racing NSW, with the addition of local rules of racing. Australian Rule (AR) 137A deals specifically with the design and use of whips. Under the current whip rules (in place since 2009), the general sub-rule provides that ARB Stewards may penalize any rider who in a race, official trial, jump-out or trackwork, or elsewhere uses his whip in an excessive, unnecessary or improper manner (AR 137A(3)). Beyond this sub-rule, the detailed rules concerning whip use provide that *in the final 100 metres of a race, a rider may, subject to the other requirements of this rule, use his whip at his discretion, subject to the other requirements of this rule* (AR 137A(5)(b)). In addition, the Stewards may penalize any rider who in a race, official trial or jump-out uses his whip:

- *forward of his horse's shoulder or in the vicinity of its head; or*
- *using an action that raises his arm above shoulder height; or*
- *when his horse is out of contention; or*
- *when his horse is showing no response; or*
- *after passing the winning post; or*
- *causing injury to his horse; or*
- *when his horse is clearly winning; or*
- *has no reasonable prospect of improving or losing its position; or*
- *in such manner that the seam of the flap is the point of contact with the horse, unless the rider satisfies the Stewards that this was neither deliberate nor reckless. (AR 137A(4))*

The last of these elements is presumably difficult to police because one has to be very close to the whip to know exactly how it is aligned with the horse's skin at the time of impact. It is of interest because the padded whip has been promoted as being the solution to welfare issues surrounding whip use. The ARB stipulates that certain designs of padded whips for use in Australian racing comply with the following specifications [6]:

1. *Maximum length of whip not exceeding 70 cm.*
2. *No binding within 18 cm of end of whip.*
3. *Minimum diameter of 2.5 cm.*
4. *Minimum length of pad not less than 18 cm for whips 60 cm in length or for longer whips the flap length must not be less than 30% of the whip length.*
5. *The overall weight of the whip must not exceed 160 gm.*
6. *The contact area of the shaft (including pad) must be smooth with no protrusion or raised surface.*
7. *The padded segment of the whip shall consist of a material approved by Stewards that does not harden over time. Padded materials must be waterproof.*
8. *Leather pads are not permitted.*
9. *The inner padded section shall consist only of closed cell foam of not less than 7 mm (one side) in thickness.*
10. *The thickness of the outer pad can be no greater than 1 mm.*
11. *All whips must be dark in colour.*

Of these, point 6 is of particular interest since it refers to the “contact area of the shaft (including pad)”. This implies that despite all the effort expended in padding the whip, there remains an expectation that the shaft will make contact in unpadded sections.

The padding is intended to absorb the impact of the whip as it makes contact. However, several images have recently come to the attention of the Australian press showing the whip indenting the skin of the horse [7]. The question of how common such visible indentations are is therefore topical.

The British Horseracing Authority (BHA) makes the stipulation that backhand position refers to a rotation that is clockwise from the jockey's perspective [8]. There are no stipulations about how the whip is to be gripped by the jockey's hand, although this is subject to some variation and may have implications for horse comfort and welfare.

The distinction between forehand and backhand whip action is critically important under the ARB's Rules of Racing [9]. The ARB rule AR137A(5)(a) states that prior to the 100 m mark (i.e., 100 m from the winning post):

- *The whip shall not be used in a forehand manner in consecutive strides.*
- *The whip shall not be used in a forehand manner more than on 5 occasions.*
- *The rider may at his discretion use the whip with a slapping motion down the shoulder, with the whip hand remaining on the reins, or alternatively in a backhand manner.*

The Stewards of Australian Racing view head-on footage retrospectively to police these rules. In contrast, the current study explored opportunistic high-speed footage of the ends of 15 races that could be viewed frame-by-frame to examine the area struck, the percentage of unpadded section making contact, whether the seam was making contact, and whether a visible indentation was evident on impact. It also recorded: the use of clockwise or counter-clockwise arm action; each of the jockeys' whip grip (i.e. whether the whip was held like a tennis racquet or a ski pole); whether the hindleg on the side of the impact was in stance or swing phase; and whether the jockey's arm was seen traveling above shoulder height.

## Materials and Methods

High-speed footage of horses galloping at two meetings from Gosford Racecourse in NSW was obtained opportunistically and analyzed under the University of Sydney's Human Research Ethics Committee (approval number 11-2009/12299). The high-speed camera was mounted at the level of the grandstand and was fixed to cover the last 200 meters of the race. The lead horse was followed as the group ran by.

Approximately the final 200 meters of each race was videoed from the left side of the horses. Analysis of the saddlecloth numbers and the horses' placings confirmed that the meetings were at the same track on the 9^th^ (races 1, 2, 3, 5, 6, 7 and 8) and the 24th June 2011 (the first 8 races). The last race at the first meeting and the last two races at the second meeting were not recorded, perhaps due to failing light. Race 4 in the first meeting was also absent but it is not clear why. The meetings were not exceptional in that they were not part of a particular racing carnival with premium prize money, so it was expected that the findings would be representative for whip use more generally in Australia. The two meetings had slightly different track conditions: “dead” on the 9^th^ and “slow” on the 24th. Prize money for all recorded races was $10,400 for first place, $3,200 for second and $1,600 for third. The average distances, numbers of starters and official times for all recorded races were 1433 meters, 10.6 runners and 89 seconds, respectively.

Inspectors from RSPCA NSW were present at this track on the 24^th^ June. In the 15 recorded races, Racing NSW Stewards reported only one violation of the whip rules. On the 24^th^ June 2011, “*Apprentice S Lisnyy, the rider of Allandy [in Race 7], was fined $100 under Rule 137A(4)(c) for use of the whip when his mount was out of contention*”.

All arm and wrist actions (n = 350) that led to whip movement on the left side of the horse were analysed by two observers (both of whom hold higher degrees in animal behavior) working independently after being instructed to follow each horse as identified by its saddle cloth and who were blinded to the other observer's scores. These actions were made by a total of 23 jockeys on 40 horses. The timing of each action was recorded as the whip made contact or, in the cases of non-contact sweeps, as it reached the descent of its arc along the horse's body. Each whip strike was identified by the time of impact, according to the time stamp on the video file. We excluded any whip use that was recorded by either observer as obscured. Reasons for obscuring included: obscured by other horses (n = 81), obscured by infrastructure such as posts or signage (n = 4), obscured by the saddlecloth (n = 10), blurred or in shadow (n = 7) or out of frame (n = 8). Actions that led to impacts in two horses on the head (n = 10), ears (n = 3) and neck/shoulders (n = 12) as recorded by both observers were also not examined further. SPSS v18 (IBM Statistics, USA) was used to conduct a pair-wise correlation on the two observers' scores of whip impacts to measure inter-observer reliability.

We used frame-by-frame analysis to examine the area struck (percentile of whip that made contact with the abdomen, percentile of whip that made contact with the hindleg or if the whip struck another area of the body). We removed head, ear, neck and shoulder impacts because whipping behind the saddle is the focus of Australian rules surrounding whip use. The remaining clear impacts on areas of the horse behind the saddle (n = 109) then formed the focus of our analysis. They all were the result of arm actions rather than wrist actions. For these, we scored the percentage of unpadded section making contact (ten percentiles); whether the seam of the padded section of the whip was making contact (Y/N or ?); and whether a visible indentation was evident on impact (Y/N or ?). We also recorded the use of clockwise or counter-clockwise arm action from the jockeys' perspective (CW or CCW); each of the jockeys' whip grip (whether the whip was held like a tennis racquet, a ski pole, or using an intermediate grip: racquet, ski-pole or intermediate); whether the hindleg on the side of the impact was in stance or swing phase (swing or stance); and whether the jockey's arm was seen travelling above shoulder height (Y/N).

Also listed was the incidence of whip impacts where both observers had recorded 100% of the abdomen or >10% of the abdomen being hit. Attributes of specific whip strikes (as specified by the time of impact) for which there were discrepancies between the scoring of Observer A and Observer B were excluded from the total. This gave the number of specific whip strikes on which both observers agreed. By dropping discrepancies, we corrected the data so that inter-observer reliability was not an issue.

## Results

**Figure 1:**
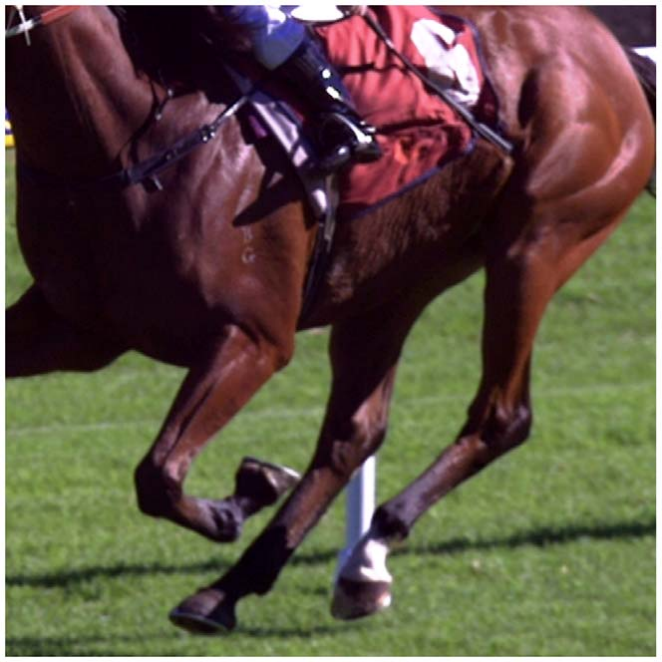
An example of the indentations occurring in more than 80% of the impacts reported by the two observers for the 109 actions that resulted in an impact behind the saddle.

From a total of 350 rider/horse interactions, there were 134 whip impacts and 104 sweeping actions that did not make contact. After removing impacts in areas not behind the saddle, 109 clear impacts were identified (see example in Fig. 1). These actions were made by a total of 21 jockeys on 31 horses. The attributes of fully recorded whip impacts are summarized in Table 1.

**Table 1:** Occurrence of each attribute reported by the two observers for the 109 actions that resulted in an impact behind the saddle.

Attribute	Observer A	Observer B	Number of specific whip strikes on which both observers concurred	Concurring results as percentage of all impacts
Only the unpadded section of the whip made contact	20	10	4	3.66
>10% of the unpadded section of the whip made contact	92	74	70	64.22
Visible indentation on impact	98	102	90	82.57
Seam was the point of contact	1	11	1^a^	0.91
Only the abdomen struck	38	36	34	31.19
>10% of the impact struck the abdomen	94	85	82	75.23
Only the hindleg struck	18	24	15	14.76
>10% of the impact struck the hindleg	73	73	71	65.13
Clockwise whip use	13	8	3	2.75
Counterclockwise whip use	96	101	91	83.48
Whip grip – Racquet	22	23	20	18.35
Whip grip – Ski pole	78	78	76	69.72
Whip grip – Intermediate	9	8	8	7.34
Left hindleg in swing phase	107	109	107	98.17
Arm traveled above the shoulder	17	19	14^b^	12.84

These data were drawn from videos taken from the outside of the track in 15 races over two meetings at the same clockwise racetrack. Unless otherwise stated, these data represent information on 21 jockeys and 31 horses.

a Observer A was unable to make a definitive decision on 99 occasions and Observer B on 30 occasions.

b Data on 7 jockeys riding 7 different horses.

### Inter-observer reliability

Pearson's correlation coefficients between observers' scores for percentage of the abdomen struck, percentage of the hindleg struck and percentage of the unpadded section making impact were r = 0.791 (P<0.001), r = 0.973 (P<0.001), and r = 0.217 (P = 0.023), respectively.

For data on arms seen traveling above the shoulder, the Pearson's correlation coefficient was r = 0.766 (P<0.001). However, for seam contact they were r = 0.017 (P = 0.861) and visible indentation r = 0.077, (P = 0.430). For the hindleg phase attribute (swing versus stance), there was insufficient variation for correlation testing to be of use.

## Discussion

The putative expectation that the shaft will make contact in unpadded sections has been borne out by the current findings. In general, unpadded sections of the whip are more likely than not to make contact with the horse. These data include contact made by the binding that fixes the padded flap to the shaft of the whip. Previous research has indicated that such binding (which in itself cannot be padded) can increase the damage potential of whips [10]. This is an extremely important finding given the racing industry's description of the padded whip as the pain-free whip [11]. The padded whip is offering less protection than might be expected in well over half of all strikes. It must be said that, being closer to the jockey's hand, the unpadded sections are therefore traveling more slowly than the tip of the whip and so presumably can do less damage on impact. However, being close to the jockey's hand means that the unpadded section is also more likely to strike the abdomen than the hindleg (see later). This is troubling, given that the skin of the abdomen is thought to be particularly sensitive [12]. Until the entire length of the shaft of the whip is padded, the racing industry's reliance on this device as a means of assuring horse welfare must be questioned.

The current findings suggest that it is normal for the padded whip to make visible indentations. Indentations occurred in more than 80% of the impacts in the current sample. Comparative studies in mice and humans have shown that deformation of the skin is likely to be detected by cutaneous nociceptors [13]. However, equine data on cutaneous nociception are not available so, it is not presently known if such stimuli are noxious in racing horses, nor is it clear if inflammation occurs as a result of repeated use of the whip. However, if the whip does lead to nociceptor activation and if inflammation does occur under these circumstances, then concerns about the adequacy of current monitoring of whip use during races are warranted.

Arguably, the prospect of a share in the prize money may incite jockeys to hit horses hard. These meetings were at a provincial track on days without exceptionally large prize money. Therefore, it is unlikely that jockeys were more motivated than usual to strike their horses. This study has produced evidence that indentations such as that depicted in Fig. 1 are common. A scoring system for the severity of indentations would be an ideal means of reporting this quality of whip impact. However, given the current imaging system and concerns about concordance between the current observers using a binary system of reporting indentations, this seems ambitious.

The current finding of impact from the unpadded shaft of the whip is also of concern. Again, data specific to horses are not available. A previous study on cats involving using an unpadded flexible rod to deliver a noxious stimulus showed that cutaneous and probably deeper (muscle) nociceptors were acutely activated and that repeated application reduced the thresholds for nociceptor activation and enhanced the duration of their responses [14]. Any extrapolation from the rigid rod used on the cats to the unpadded shaft of whip used on racing horses is speculative. But again, if the whip does lead to nociceptor activation in horses and if inflammation does occur under racing conditions, the current finding of impact from the unpadded shaft of the whip is of concern.

Inflammation is well documented to reduce the thresholds for nociceptor activation and to enhance the duration of their responses (for review, see [15]). Whether nociceptor activation results in pain perception is therefore a critical issue to be considered. If, akin to the often cited “battlefield analgesia”, the context of a race constitutes a “stressful” environment and stress-induced analgesia is invoked to defend the use of a stimulus with the potential to produce pain, then as identified in a recent letter to the editor of the journal Pain [16], there is a critical requirement for empirical evidence supporting this view. If there is an anti-nociceptive/analgesic state during the race, it begs the question that if the stimulus (the whip) has lost its salience, then why use it at all? The need for empirical evidence is highlighted further, given recent evidence that there is enhanced, rather than reduced pain perception characterizing certain distinct stress-related, affective states (e.g. [17], [18], [19], [20]).

Comparative studies suggest that repeated noxious stimuli may result in ongoing reductions in the thresholds for activation of both mechano-, thermo-, and nociceptive stimuli [14], [21], a phenomenon first described in rats by Lewis more than 75 years ago [22]. The increased sensitivity of the receptors leads to enhanced pain perception or even touch-evoked pain, and are understood as adaptive responses which direct an organism's behavior to recovery mode, itself a critical component of the pain-injury response continuum [23], [24]. It has been observed both clinically and experimentally that such changes in pain perception can in certain circumstances persist (for review, see [25]). Thus the post-race impact of whip use must form an important part of the empirical evaluation of the painful effects of whip use.

The indentations around the whip strongly imply that the seam of the padded section often makes contact with the horse's skin. However, on this matter, the rules of racing are concerned only with the seam being the point of contact and even then only when such contact was either deliberate or reckless. This presumably means that the Stewards are concerned for the welfare of horses if the seam, which is more resilient than other parts of the padded whip and therefore more likely to damage the skin, is the *first* part of the whip to strike the horse. Despite working with high-resolution files (each 200 m section amounted to approximately 260 Mb) that had captured approximately 1000 frames per whip cycle, in more than 99% of cases, it was not possible to see if the seam was the first point of contact that the whip made with horse's skin. Given our difficulty, this information must be extremely difficult, if not impossible, to discern using the Stewards' high-definition video footage, which operates to a maximum of 25 frames per second. This may explain why the number of times breaches of the seam contact rule have ever been prosecuted since the current rules were established is negligible. Another explanation is that it may be difficult for the Stewards to adequately demonstrate that any visible seam contact has been deliberate or reckless. In the current study, the seam was considered the point of contact only once. All other strikes were inconclusive. Therefore, it is suggested that the seam rule is virtually impossible to police, even using significantly more detailed footage than is usually reviewed by racing Stewards, and its inclusion is therefore futile.

The two horses that were struck on the head and ears may be anomalies among this dataset but they are also concerning, since they appear to represent a breach of the rules surrounding whip use. These incidents should perhaps have been commented upon by the Stewards but the quality of the footage they review may prevent analysis of the standard reported here. One of the horses in question was being ridden by a jockey who had stowed his whip in his right hand with a racquet grip and had pushed his hands right up the horse's neck as he rode it home. Therefore, rather than being wielded, the whip was flapping against the horse's head and ears. The second horse was struck on the padded cheek-piece of the bridle. It is not clear whether this contact with gear would be of interest to the Stewards in the spirit of the rule about whip use forward of the horse's shoulder or in the vicinity of its head.

The observers in this study agreed that the abdomen is twice as likely to be struck with the entirety of the whip than is the hindleg. This suggests that the abdomen rather than the leg is being targeted. In its discussion of appropriate use of the whip, the Horse Racing Authority (HRA, later to become the British Horseracing Authority) [26] stated that “*whips should only be used on the quarters with the whip in either the backhand or forehand position or down the shoulder with the whip in the backhand position*”. In veterinary nomenclature, the abdomen is not regarded as part of the quarters. In effect, only a quarter of the whip strikes in this study would be permitted under UK whip rules. It is interesting that the quarters are not specified in the Australian Rules of Racing. There is evidence from general equitation texts that the inguinal and abdominal regions are particularly sensitive to tactile stimulation [11], so in any attempt to optimize the welfare of horses being whipped, it would be good to see more guidance to spare horses from impact in these areas.

Arm action that was counter-clockwise from the jockey's perspective was far more common than clockwise arm action. According to HRA stipulations, this suggests that backhand whipping is uncommon. However, this stipulation is clearly flawed since the hand in which the whip is being held will influence whether it appears to travel clockwise or counter-clockwise when viewed from the jockey's perspective. The counter-clockwise actions in the left hand, as reported here, are associated with backhand whip use and clearly predominate. With the strong focus on forehand action in the current Australian whip rules, it is possible that the rules have inadvertently encouraged jockeys to use backhand rather than forehand actions to avoid being penalized. The preferred grip was holding the whip like a ski pole. This is diagnostic of backhand whip use and therefore confirms the predominance of a whip action that largely grants immunity from the rules that limit the number of whip strikes and the use of the whip with consecutive strides prior to the 100 m mark (i.e., 100 m from the winning post). These data suggest that the current focus on forehand whip use has lost some relevance

In this study of 109 impacts, the jockey's arm was seen traveling above shoulder height on 14 occasions. It is not clear how much, if any, amplification in force that whipping from this height creates. From the perspective of force and therefore pain, the recoil of the whip may be more important than the height from which it descends during its trajectory. Certainly, the bend in the whip was considerable in many cases and this suggests stored energy that will be released on impact with the horse. In almost all cases, the hindleg on the side of the impact was in the swing phase when the whip made contact with the horse. This aligns with good practice in equitation science [27] since, in contrast with cueing the horse to respond while its leg is supporting its bodyweight, this technique is thought to increase the chances of the horse being able to respond promptly.

This study reports only left-handed whip use on 40 horses by a total of 23 jockeys on a counter-clockwise course. It may be that right-handed whip use differs, which is especially relevant since a previous study has shown that the right hand is generally favored by Australian jockeys [5]. A larger study may be needed to explore the extent to which these findings reflect whip use across Australia and internationally. It would be useful to see high-speed cameras used for all such studies. Despite the quality of the images derived from the high-speed camera in the current study, inter-observer reliability was only moderate for seam contact and visible indentation, reflecting the difficulties in making definitive observations, especially when horses are furthest from the camera.

There was evidence of at least 28 examples, in 9 horses, of breaches of the whip rules (one seam contact, 13 contacts with the head, and 14 arm actions that rose above the height of the shoulder). There will be differences between what is reported here and the official version of whip use at these two meetings, not least because Racing NSW Stewards review footage recorded at fewer frames per second and also because they view head-on footage. Head-on footage is preferred by Stewards because it allows estimations of whip use on both sides of the horses. However, for examination of details such as seam contact, proportion of abdomen struck and whip use *forward of the horse's shoulder or in the vicinity of its head*, it may be less useful than the lateral footage used in the current study.

None of the breaches detected herein was reported by the Racing NSW Stewards. The only one violation of the whip rules Racing NSW Stewards reported from the recorded 15 races, was not recorded in the current series of arm and wrist actions because the horse finished in tenth place and so was outside the current study's focus which was on footage of the leading horses in each finish. The limitations on the ability of Stewards to detect and therefore report breaches may raise concerns about horse welfare if the whip rules are intended to ensure that horses are not whipped unnecessarily. A similar standard is also employed in animal welfare legislation in the other states. Ideally, high-speed footage from both sides of the horses should be used in any optimal bid to enforce the rules and safeguard horse welfare as proposed by the ARB [28]. That said, some impacts will even then be obscured by other horses, and occasionally by infrastructure, as was frequently the case in the current study.

High-speed footage of horses at the end of races reveals numerous unreported breaches of the rules surrounding whip use and the unpadded section of the whip making contact on 64% of impacts. These results cast significant doubt on the current ability of Stewards to effectively police the rules concerning whip use and, more importantly, challenge the notion that padding the distal section of the whip completely safeguards horses from any possible whip-related pain.
